# *Tbx5* Buffers Inherent Left/Right Asymmetry Ensuring Symmetric Forelimb Formation

**DOI:** 10.1371/journal.pgen.1006521

**Published:** 2016-12-19

**Authors:** Fatima A. Sulaiman, Satoko Nishimoto, George R. F. Murphy, Anna Kucharska, Natalie C. Butterfield, Ruth Newbury-Ecob, Malcolm P. O. Logan

**Affiliations:** 1 Division of Developmental Biology, MRC-National Institute for Medical Research, Mill Hill, London, England; 2 Randall Division of Cell and Molecular Biophysics, King’s College London, Guy’s Campus, London, England; 3 Dept of Plastic & Reconstructive Surgery, Great Ormond St Hospital for Children NHS Foundation Trust, London, England; 4 Department of Clinical Genetics, University Hospitals Bristol NHS Foundation Trust, St. Michaels’s Hospital, Bristol, United Kingdom; Stanford University School of Medicine, UNITED STATES

## Abstract

The forelimbs and hindlimbs of vertebrates are bilaterally symmetric. The mechanisms that ensure symmetric limb formation are unknown but they can be disrupted in disease. In Holt-Oram Syndrome (HOS), caused by mutations in *TBX5*, affected individuals have left-biased upper/forelimb defects. We demonstrate a role for the transcription factor *Tbx5* in ensuring the symmetric formation of the left and right forelimb. In our mouse model, bilateral hypomorphic levels of *Tbx5* produces asymmetric forelimb defects that are consistently more severe in the left limb than the right, phenocopying the left-biased limb defects seen in HOS patients. In *Tbx* hypomorphic mutants maintained on an *INV* mutant background, with *situs inversus*, the laterality of defects is reversed. Our data demonstrate an early, inherent asymmetry in the left and right limb-forming regions and that threshold levels of *Tbx5* are required to overcome this asymmetry to ensure symmetric forelimb formation.

## Author Summary

Externally, the human form appears bilaterally symmetric. For example, each of our pairs of arms and legs are the same length. This external symmetry masks many asymmetries found in internal organs. In most people the heart is found on the left side of the chest. The stomach, liver and spleen are also positioned asymmetrically. The authors of this study demonstrate, using a mouse model, that bilateral symmetry of the arms is not a default, passive state but that mechanisms are in place that ensure symmetrical formation of the left and right limbs. Bilateral symmetry of the arms is achieved by the action of a gene *Tbx5* that masks the effects of signals that acted earlier during embryogenesis, many days before limb formation, and imposed asymmetries on the forming internal organs. Maintaining bilateral symmetry of the arms is important for them to carry out their normal functions but this process can go wrong. Holt-Oram syndrome patients have upper limb defects, including shortened arms. Consistently the defects are more severe in their left arm than right. This birth defect is caused by disruption of the *TBX*5 gene. By linking the action of Tbx5 to symmetrical limb formation, the authors provide an explanation for why Holt-Oram syndrome patients have more severe defects in the left arms than right.

## Introduction

The external body plan of most metazoans is bilaterally symmetric. How this symmetry is achieved has fascinated biologists for centuries and is exemplified by Leonardo Da Vinci’s description of the “Vitruvian Man” that describes some of the uniform proportions of the human body. In vertebrates, external bilateral symmetry masks significant internal asymmetries such as the position of the heart and liver and number of lobes of the lungs. While there has been progress in identifying how asymmetry of internal organs is generated [1], we know very little about how symmetry in bilateral structures, such as the limbs is established. A classic study that compared the lengths of the skeletal elements of left and right embryonic chick wings found there was very little difference in their size [2] demonstrating the exquisite fidelity in the genetic programmes regulating limb development, a finding all the more intriguing by the lack of evidence for molecular crosstalk between the developing left and right limb buds.

Holt Oram Syndrome (HOS) [OMIM 142900] is caused by mutations in *TBX5*, a T-box transcription factor expressed in the forelimb and heart. The clinical features of HOS include heart defects and a spectrum of upper/fore limb defects ranging in severity from total aplasia of the radial elements to relatively mild defects such as a tri-phalangeal thumb [3]. A characteristic feature of HOS patients is that although the severity of limb defects can vary between patients, the left limb is more severely affected than the right in most patients [3]. HOS is a dominant disorder and heterozygous affected individuals carry mutations in *TBX5* predicted to result in truncated proteins that fail to fold or are rapidly degraded [4] and are most likely loss-of-function alleles. HOS defects are therefore thought to arise as a result of TBX5 haploinsufficiency and indicate that both copies of the gene are required for normal function.

Attempts to recapitulate the upper limb defects associated with HOS in the mouse have previously been unsuccessful. Conditional heterozygous deletion of *Tbx5* using the limb-restricted *Prx1Cre* (*Tbx5*^*lox/+*^*;Prx1Cre*) does not produce any obvious forelimb phenotype, while homozygous conditional deletion of *Tbx5* (*Tbx5*^*lox/lox*^*;Prx1Cre*) leads to a total loss of forelimb bud initiation and subsequent forelimb formation [5]. We have developed two alternative strategies to model the left-biased, asymmetrical limb defects of HOS patients in the mouse. One strategy utilizes a gene deletion-gene replacement strategy [6] in which *Tbx5* is conditionally deleted using the *Prx1Cre* line and hypomorphic levels of a *Prx1-Tbx* transgene are expressed in the forelimb regions of the same embryo. The alternative strategy uses a mosaic cre line, *Prx1Cre(98)*, to conditionally delete *Tbx5* from cells in the right and left forelimb-forming LPM. Both approaches successfully recapitulate the types of limb abnormalities observed in HOS patients and significantly, the left bias in the severity of the limb defects. We also demonstrate that the disruption of *INV*, a gene crucial for the establishment of the left-right pathway [7] in the presence of hypomorphic levels of *Prx1-Tbx* lead to the formation of right sided forelimb defects. Additionally, optimal levels of *Fgf10* expression in the presence of hypomorphic *Prx1-Tbx* levels are not sufficient to rescue symmetrical forelimb bud initiation and formation. These data demonstrate that although the left and right limb ultimately develop to be bilaterally symmetric structures they arise from regions of the left and right embryo flank that have inherent asymmetries. We further show that threshold levels of *Tbx5* are required to overcome these asymmetries to ensure bilaterally symmetric forelimb formation.

## Results

### Hypomorphic *Tbx* levels recapitulate HOS limb defects

**Fig 1:**
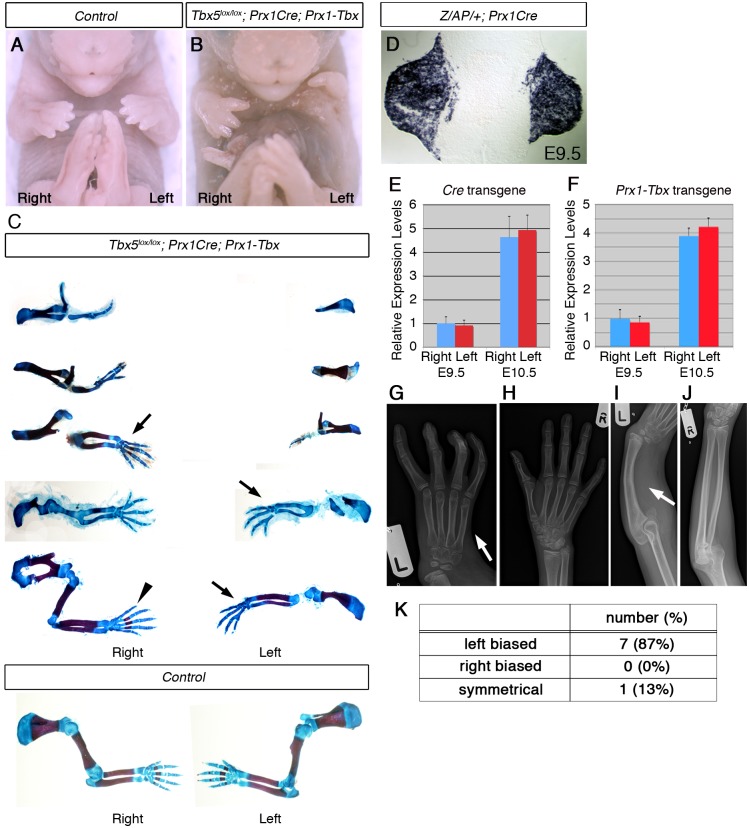
A mouse *Tbx* hypomorph mutant produces left-biased asymmetric forelimb defects. **A-B,** E17.5 control (A) and *Tbx5*^*lox/lox*^*;Prx1Cre;Prx1-Tbx* mutant (B). **C,** *Tbx5*^*lox/lox*^*;Prx1Cre;Prx1-Tbx* mutant forelimb skeletal preparations shown in descending order of severity (top to bottom) and E17.5 control forelimbs. The defects are consistently more severe in the left forelimb than the right (n = 11). Absent digit 1 (thumb) (arrows), triphalangeal digit 1 (arrowhead). **D,** A transverse section at the level of the forelimb bud of a E9.5 *Z/AP/+;Prx1Cre* embryo. **E,** qPCR analysis of left and right forelimb buds of *Prx1Cre* embryos. **F,** qPCR analysis of the left and right forelimb buds of *Prx1-Tbx* transgenic embryos. **G-J.** X-ray radiographs of a HOS patient. The left forelimb is more affected than the right one. The thumb is absent on the left hand (G, arrow) while it is present on the right (H). The radius is missing on the left side (I, arrow), while it is formed on the right (J). **K.** Numbers of patients showing left-biased, right-biased and symmetrical forelimb defects.

To generate a model mouse of HOS, we used a gene deletion-gene replacement strategy (S1A Fig) [6] so that hypomorphic levels of a *Tbx* transgene are delivered to the forelimb-forming region. Previously, we have shown that homozygous deletion of *Tbx5* using the limb-restricted Cre-deleter line, *Prx1Cre*, produces a forelimb-less phenotype [5]. This defect can be completely rescued by misexpression of either *Tbx5* or *Tbx4* transgenes driven by the same *Prx1* regulatory element [8]. One chimeric construct, *Prx1-5N5T4C#1*, a fusion of the N-terminal and T-Box DNA binding domains of Tbx5 and the C-terminal domain of Tbx4, is also able to rescue forelimb bud formation [8]. These results indicate that Tbx5 and Tbx4 have common functions in initiation of fore and hindlimb outgrowth and Tbx5, Tbx4 and Tbx5/Tbx4 chimera proteins can act equivalently. Another transgenic line, *Prx1-5N5T4C#2* (hereinafter referred to as *Prx1-Tbx*), harbours the same transgene as *Prx1-5N5T4C#1* but in a different locus and expresses hypomorphic levels of the chimera protein compared to endogenous *Tbx5* (S1B–S1E Fig). This hypomorphic misexpression of the transgene only partially rescues forelimb bud initiation defects caused by the conditional deletion of *Tbx5* (*Tbx5*^*lox/lox*^*;Prx1Cre;Prx1-Tbx*) and recapitulates many features of the upper limb defects seen in HOS patients, for example an anterior bias to the structures affected (including triphalangeal thumb, absent thumb), defects in the scapula and in more severe examples, phocomelia (Fig 1A–1C). Another clinical feature of HOS is the broad range in the severity of limb defects, even within a family carrying the same *TBX5* mutation, ranging from almost complete absence of the upper limb to triphalangeal thumb. This is also reproduced in our mouse model (Fig 1C). Despite the range in the severity of the limb defects between different embryos, in individual *Tbx5*^*lox/lox*^*;Prx1Cre;Prx1-Tbx* mutant embryos the forelimb defects are consistently more severe in the left forelimb than the right. In our mouse model this was observed in 100% of the embryos studied (n = 11) (S2 Table). A transverse section of *Z/AP/+;Prx1Cre* embryo shows the bilaterally symmetrical Cre activity in the left and right forelimb buds [9] (Fig 1D). Furthermore, we compared *Cre* mRNA expression levels between the left and right forelimb buds and did not detect statistically significant difference (p > 0.05 with two-tailed Student’s t-test) (Fig 1E). This result is consistent with the fact that the *Prx1Cre* driver has been used in combination with numerous other conditional mutant alleles and all limb defects reported to date are bilaterally symmetric. In addition, equivalent, bilateral chimera transcript levels are present in *Prx1-Tbx* transgenic embryos (p > 0.05 with two-tailed Student’s t-test) (Fig 1E), indicating Tbx activity that partially and asymmetrically rescues limb outgrowth is bilaterally symmetric in *Tbx5*^*lox/lox*^*;Prx1Cre;Prx1-Tbx* mutant embryos.

In a previous study it was calculated that 70% of HOS individuals display asymmetrical upper limb defects, and in 91% of these, the left side is more severely affected than the right [3]. In contrast, 100% of the Tbx hypomorphic mutant mice we obtained showed left-biased defects. A possible explanation for this discrepancy in penetrance is that the original analysis of HOS patients was carried out before genetic lesions in *TBX5* were identified as responsible for HOS and some of the original patients may not have had pathogenic *TBX5* mutations. We searched the electronic patient record at Great Ormond St Hospital and identified 32 patients with a clinical suspicion of Holt-Oram syndrome. In 11 cases *TBX5* mutations had been identified, 8 of which were pathogenic mutations (S1 Table). 7 cases show left-biased forelimb defects and one case is symmetrical (Fig 1G–1K) [10–13]. We did not observe right-biased defects in any patients with confirmed pathogenic *TBX5* mutations. Therefore in our patient series when pathogenic mutations in *TBX5* have been confirmed the clinical presentation includes left-biased severity with almost 100% penetrance consistent with what we observed in our mouse model.

**Fig 2:**
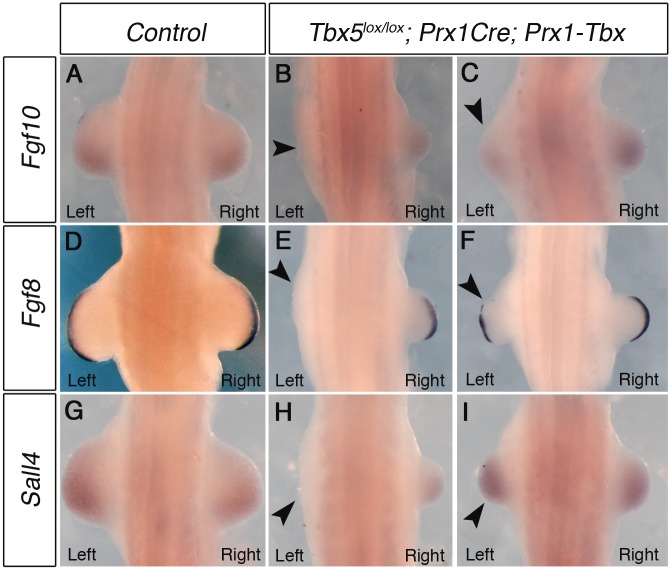
Marker gene expression in left and right forelimb buds of *Tbx* hypomorphs. WISH analysis of E10.5 embryos. **A,** *Fgf10* expression in a control embryo **B,** Left forelimb bud is not formed and *Fgf10* expression is lost on the left side (arrowhead) in a severely affected embryo **C**, *Fgf10* is weaker in left forelimb of a mildly affected embryo (arrowhead). **D,** *Fgf8* expression in control **E**, *Fgf8* is absent in left AER of a severely affected embryo (arrowhead). **F,** *Fgf8* is disrupted in left AER of a mildly affected embryo (arrowhead). **G,** *Sall4* expression in control. **H**, *Sall4* is absent in left LPM of a severely affected embryo (arrowhead). **I**, *Sall4* is expressed at similar levels bilaterally in a mildly affected embryo. A minimum of 4 mutants for each phenotype were analysed with each probe.

During forelimb bud initiation, *Tbx5* induces the expression of *Fgf10* in the limb mesenchyme and *Fgf10* subsequently induces *Fgf8* expression in the overlying ectoderm. *Sall4* is also proposed as a target of *Tbx5* [14,15]. We examined whether the expression patterns of these genes are different between the right and left forelimb buds of *Tbx5*^*lox/lox*^*;Prx1Cre;Prx1-Tbx* mutant embryos. At E10.5 mutant embryos can be divided into two groups depending on the severity of phenotypes, which correlate with the variation in forelimb defects observed later at E17.5. In the first group, there is no obvious left limb bud and small right limb buds are formed (Fig 2B, 2E and 2H). *Fgf10*, *Fgf8* and *Sall4* are not detected in the left lateral plate mesoderm (Fig 2B, 2E and 2H arrowheads). In contrast, low levels of *Fgf10* and *Sall4* are detectable in the right forelimb buds and *Fgf8* is present in the right AER. In this group of mutants the left limb bud lacks the first emergence of the bud, rather than a subsequent regression. In the second group, they form small left limb buds (Fig 2C, 2F and 2I, arrowheads). The right limb buds are larger than left ones but still smaller than control limb buds. *Fgf10* expression in the left forelimb bud is lower than the right (Fig 2C), whereas there is no detectable difference in *Sall4* expression between the left and right forelimb buds (Fig 2I). *Fgf8* expression in the left AER is disrupted in contrast to the right AER where it is expressed throughout (Fig 2F).

Together, these results demonstrate that hypomorphic levels of a *Prx1-Tbx* transgene cause the limb outgrowth defect by failing to establish the correct positive feedback loop of *Fgf10* and *Fgf8* and this defect is consistently more severe in the left forelimbs than the right.

### Mosaic *Tbx5* deletion causes left-biased forelimb defects

**Fig 3:**
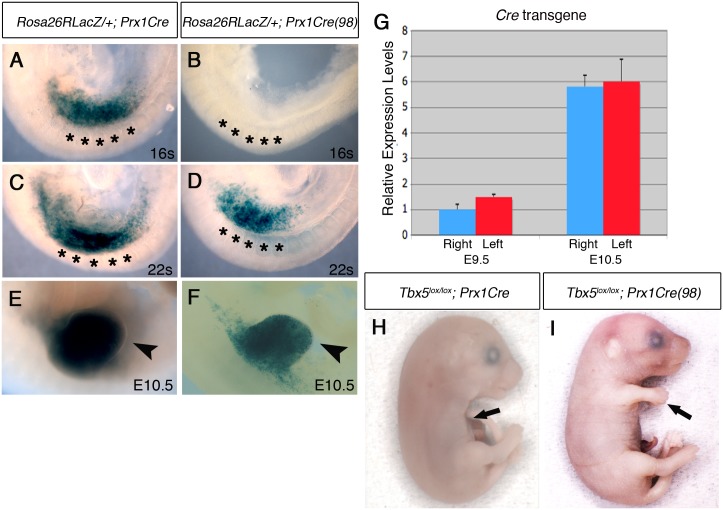
Comparison of Cre activity in *Prx1Cre* and *Prx1Cre(98)* transgenic deleter lines. **A-F**, Lateral views of right side of embryos are shown. LacZ staining of *Rosa26RLacZ/+;Prx1Cre* embryos (A, C and E) and *Rosa26RLacZ/+;Prx1Cre(98*) embryos (B, D, and F). In the *Prx1Cre* line, Cre is active throughout the forelimb-forming region (marked by black asterisks in the adjacent somites) by 16 somites stage (A) while cre activity is not detected in *Rosa26RLacZ/+;Prx1Cre(98)* at this stage (B). At 22 somites stage, staining is seen throughout the nascent forelimb bud as well as the LPM rostral and caudal to the forelimb-forming region in *Rosa26RLacZ/+;Prx1Cre* embryos (C). At this stage cre is active in the nascent forelimb bud of *Prx1Cre(98)* embryo in a ‘salt and pepper’ mosaic manner (D). At E10.5 strong staining is observed throughout the forelimb buds of *Rosa26RLacZ/+; Prx1Cre* (E, arrowhead) and in a mosaic manner in the forelimb buds of *Rosa26RLacZ/+;Prx1Cre(98)* embryo (F, arrowhead). **G,** qPCR analysis of left and right forelimb buds of *Prx1Cre(98)* embryos. Cre mRNA is expressed at a similar level on both sides. **H**, All the elements of the forelimb have failed to form in E17.5 *Tbx5*^*lox/lox*^*;Prx1Cre embryo* (arrow). **I**, Abnormal forelimbs are formed in E17.*5* ^*Tbx5lox/lox*^*;Prx1Cre(98)* embryo (arrow).

We tested if mosaic deletion of *Tbx5* in the limb mesenchyme induces similar defects as hypomorphic *Tbx* mutants. We hypothesized that following mosaic deletion of *Tbx5*, only a subset of cells would express *Fgf10* so that the total amount of secreted Fgf10 available in the forelimb LPM is reduced to hypomorphic levels. For this purpose, we used the *Prx1Cre(98)* transgenic line [16]. In this mouse, the same *Prx1Cre* transgene is integrated in a different locus, resulting in a mosaic and delayed Cre recombinase activity in the early forelimb bud (Figs 3A–3F and S2). Cre activity is not detected at 16 somites stage in *Prx1Cre (98)* (Fig 3B), when Cre from *Prx1Cre* is already active (Fig 3A), but its activity is detectable in the forelimb region at 22 somites stage and at E10.5 in a mosaic manner (Figs 3D, 3F and S2). Cre activity appears to be at equivalent levels in the left and right forelimbs when analysed with a cre reporter and stained histologically (S2 Fig). Furthermore, we confirmed the symmetrical mRNA expression levels between the left and right forelimb buds (p > 0.05 with two-tailed Student’s t-test) (Fig 3G). This mosaic and delayed deletion of *Tbx5* by *Prx1Cre(98)* is sufficient to cause forelimb defects (Fig 3H and 3I).

**Fig 4:**
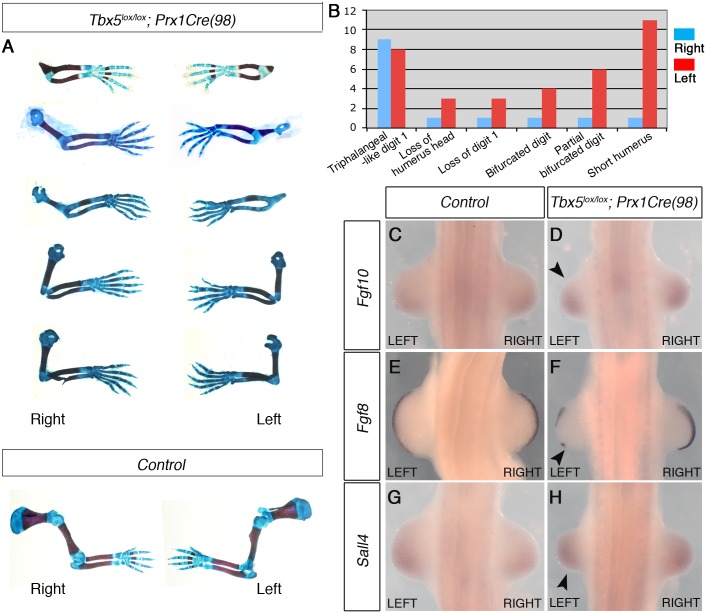
The mosaic and delayed deletion of *Tbx5* results in the formation of left-biased forelimb defects. **A**, a selection of 5 pairs of the left and right forelimbs of E17.5 *Tbx5*^*lox/lox*^*;Prx1Cre(98)* mutants and control embryos illustrating the range in forelimb defect severity. Mutant forelimbs are shown in descending order of severity from top to bottom. **B**, Tabulation of forelimb defects observed in 18 mutant embryos. Defects are observed more frequently in the left than the right forelimbs. **C-H,** WISH analysis of E10.5 embryos. Left forelimbs are smaller than right forelimbs (n = 12) (arrowheads). *Fgf8* expression is disrupted in left AER (F) while an obvious reduction of *Fgf10* (D) and *Sall4* (H) is not observed.

These *Tbx5*^*lox/lox*^*;Prx1Cre (98)* embryos display similar, although milder, defects as *Tbx5*^*lox/lox*^*;Prx1Cre;Prx1-Tbx* mutants including triphalangeal thumb, absent thumb and defects in the scapula and humerus (Fig 4A and 4B). Again, although a range in the severity of defects is observed in different embryos, consistently defects are observed with greater severity and higher frequency on the left side than the right (n = 18) (Fig 4B and S2 Table). An exception in these milder phenotypes was triphalangeal thumb that is observed with similar frequency on both sides. In mutant embryos at E10.5, forelimb buds are smaller than those of control embryos (n = 12) (Fig 4C–4H). *Fgf8* expression is disrupted on the left forelimb (Fig 4F). As the defects in this mutant are milder than those of *Tbx5*^*lox/lox*^*;Prx1Cre;Prx1-Tbx* embryos, clear reduction of *Fgf10* and *Sall4* expression patterns were not observed (Fig 4D and 4H). These results demonstrate that mosaic and delayed deletion of *Tbx5* from the left and right forelimb LPM leads to left-biased asymmetric forelimb defects.

Together, using two different genetic strategies, we demonstrate that bilateral hypomorphic levels of Tbx activity cause left-biased forelimb defects. These results reveal an inherent asymmetric difference in the left and right forelimb LPM and that an optimal level of Tbx activity is required to buffer the asymmetric difference to ensure bilateral, symmetric limb outgrowth.

### Limb-forming LPM asymmetry is linked to the left-right pathway

We tested if the inherent asymmetric difference in the left and right limb-forming LPM is downstream of the axial left-right pathway. The establishment of the left-right axis is critical for the asymmetric patterning of the visceral organs and the disruption of the left-right pathway can cause *situs inversus*, a reversal of internal organ asymmetry [17–19]. Previous studies have demonstrated that the biased asymmetry of genetic and drug-induced forelimb defects is subject to mirror reversal in *situs inversus* embryos [20,21]. Therefore, we tested whether the left bias of forelimb defects in *Tbx5*^*lox/lox*^*;Prx1Cre;Prx1-Tbx* mutants is reversed in *situs inversus* embryos. We used homozygous mutants of *INV* as the loss of this gene induces *situs inversus* rather than randomising situs as reported in the *IV* mutant [7,22,23]. We generated 30 *INV/INV* embryos, 21 of which show *situs solitus* and the other 9 embryos show *situs inversus*, indicating that in our hands and in common with other reports the penetrance of *situs inversus* in *INV/INV* embryos is lower than originally reported [7,24].

**Fig 5:**
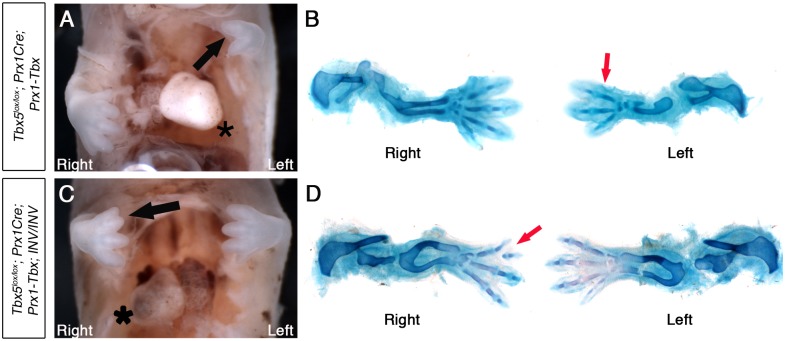
*Tbx5*^*lox/lox*^*;Prx1Cre;Prx1-Tbx;INV/INV* mutants with *situs inversus* have right biased asymmetric forelimb defects. **A,** *Tbx5*^*lox/lox*^*;Prx1Cre;Prx-Tbx* mutant embryo at E14.5. The heart is on left (asterisk). **B,** Skeletal preparation. Right forelimb has 4 digits, while left forelimb has 3 digits (red arrow). **C,** *Tbx5*^*lox/lox*^*;Prx1Cre;Prx1-Tbx;INV/INV* mutant embryo at E14.5. The heart is on the right (asterisk), indicating *situs inversus*. The right forelimb is more severely affected than the left (black arrow). **D,** Skeletal preparation. The left forelimb has 4 digits. In contrast, the right forelimb is more severely affected having 3 digits with the most anterior bifurcated (red arrow).

*Situs solitus*, or normal organ asymmetry, in E14.5 *Tbx5*^*lox/lox*^*;Prx1Cre;Prx1-Tbx* mutants is shown by the left-sided position of the heart (black asterisk, Fig 5A). In this example, the left forelimb is more severely affected (3 digits) than the right forelimb (4 digits) (red arrow, Fig 5B). Because of the low penetrance of *situs inversus* in *INV* homozygous mutants we observed, we were only able to obtain 3 *Tbx5*^*lox/lox*^*;Prx1Cre;Prx1-Tbx;INV/INV* mutants that show *situs inversus*, indicated by right-sided heart position (black asterisk, Fig 5C). In all embryos, the right limb is more severely affected (Figs 5D, S3 and S2 Table). In the embryo shown in Fig 5D the right forelimb has 3 digits with the most anterior bifurcated (red arrow) whereas the left forelimb has 4 digits.

This indicates that the left-bias of the forelimb defects caused by hypomorphic levels of the *Prx1-Tbx* transgene are reversed in embryos with *situs inversus* and embryos now display a right-sided bias in forelimb defects.

### *Tbx5* ensures symmetrical forelimb formation independent of *Fgf10*

Since *Fgf10* is a direct target of *Tbx5* during forelimb initiation, we tested whether this factor is mediating the mechanism to buffer asymmetry between the left and right forelimb-forming regions. If so, optimal levels of *Fgf10* in the *Tbx5*^*lox/lox*^*;Prx1Cre; Prx1-Tbx* mutant background would be sufficient to rescue bilaterally symmetric limb formation.

To carry out this assay, we used a Cre-inducible *Fgf10*-expressing line, *Z/EGFgf10* (see Materials and Methods). By crossing with the *Prx1Cre* transgenic, transgene-derived *Fgf10* can be expressed throughout the forelimb-forming region. First we tested the ability of the *Z/EGFgf10* transgenic to rescue *Fgf10* mutant phenotypes. *Fgf10*^*-/-*^ null mutants lack all forelimb skeletal elements as the establishment of the positive feedback loop of *Fgf10* and *Fgf8* is required for limb bud formation and subsequent outgrowth [25] (S4D–S4F Fig). *Fgf10* expression from the *Z/EGFgf10* transgene, is able to fully rescue the forelimb defects in *Fgf10*^*-/-*^ null mutants (n = 2/2) (S4G–S4I Fig and S2 Table), indicating the levels of Fgf10 produced by *Z/EGFgf10* are sufficient to support normal limb formation.

**Fig 6:**
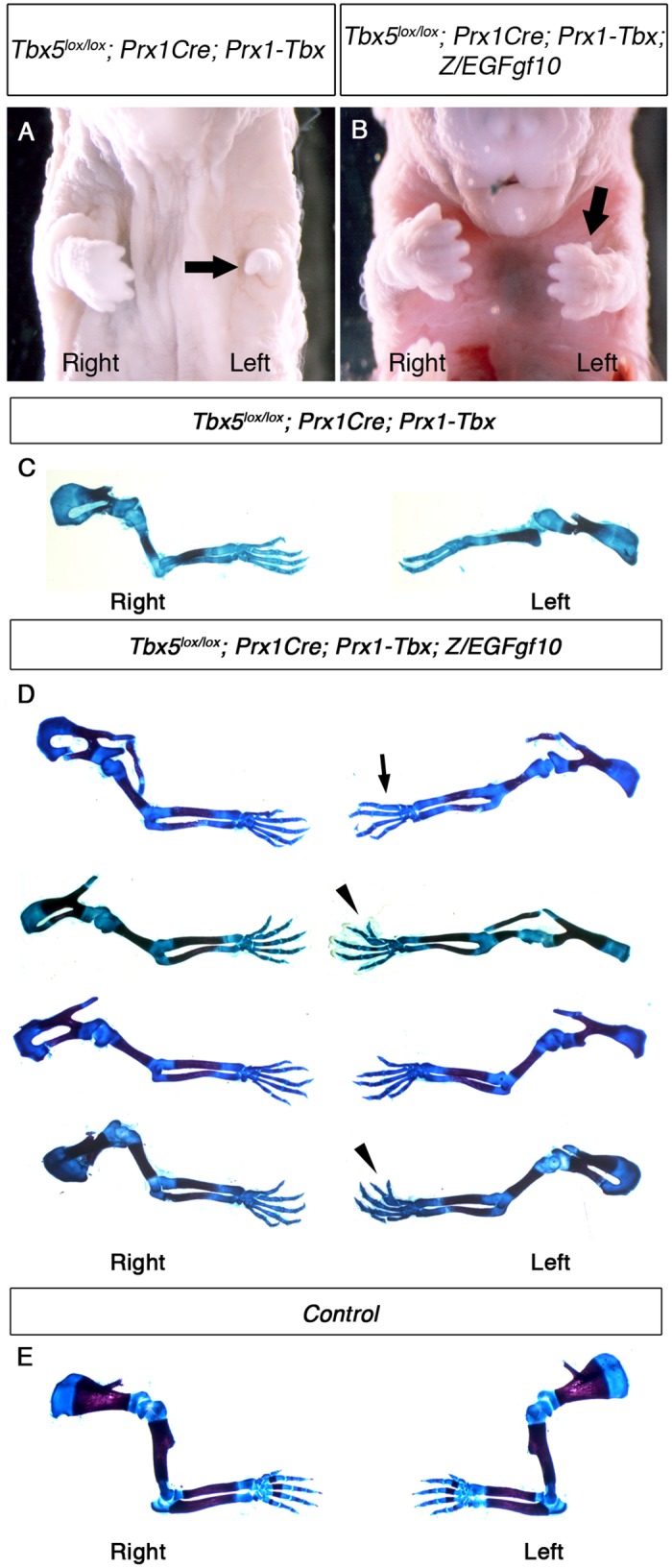
*Fgf10* expression at an optimal level in the forelimb LPM cannot rescue asymmetric defects of *Tbx5*^*lox/lox*^*; Prx1Cre; Prx1-Tbx* mutants. **A-B,** Ventral views of mutant embryos at E17.5. The left forelimb is severely truncated compared to the right in *Tbx5*^*lox/lox*^*;Prx1Cre;Prx1-Tbx* mutant (arrow) (A). *Tbx5*^*lox/lox*^*;Prx1Cre;Prx1-Tbx;Z/EGFgf10* mutant lacks digit one in the left forelimb (arrow) (B). **C-E,** Skeletal preparation of *Tbx5*^*lox/lox*^*;Prx1Cre;Prx1-Tbx* (C), *Tbx5*^*lox/lox*^*;Prx1Cre;Prx1-Tbx;Z/EGFgf10* (D) and control (E) forelimbs. Left forelimbs are more severely affected than right in both examples. Absent digit 1 (thumb) (arrow), bifurcated digit (arrowheads).

We used the *Z/EGFgf10* to test if *Fgf10* expressed from this transgene is able to rescue the asymmetric defects observed in *Tbx5*^*lox/lox*^*;Prx1Cre;Prx1-Tbx* mutants. *Tbx5*^*lox/lox*^*;Prx1Cre;Prx1-Tbx;Z/EGFgf10* mutants have milder forelimb outgrowth defects compared to *Tbx5*^*lox/lox*^*;Prx1Cre;Prx1-Tbx* (Fig 6A and 6B), consistent with previous studies showing that *Fgf10* acts downstream of *Tbx5* during forelimb initiation. Skeletal analysis of four mutants, however, demonstrates that some defects, including absent thumb, bifurcated digit, hypoplastic scapula and short humerus are still observed (Fig 6C–6E). Significantly, these defects are more severe and more frequently observed on the left side (n = 4/4) (S2 Table), indicating left-biased asymmetric differences are still present. These results demonstrate that in the presence of bilaterally symmetric, hypomorphic Tbx expression levels, raising levels of Fgf10 ligand (to a level sufficient to fully rescue limb formation in the *Fgf10*^*-/-*^ null mutants) cannot rescue symmetrical forelimb outgrowth.

## Discussion

**Fig 7:**
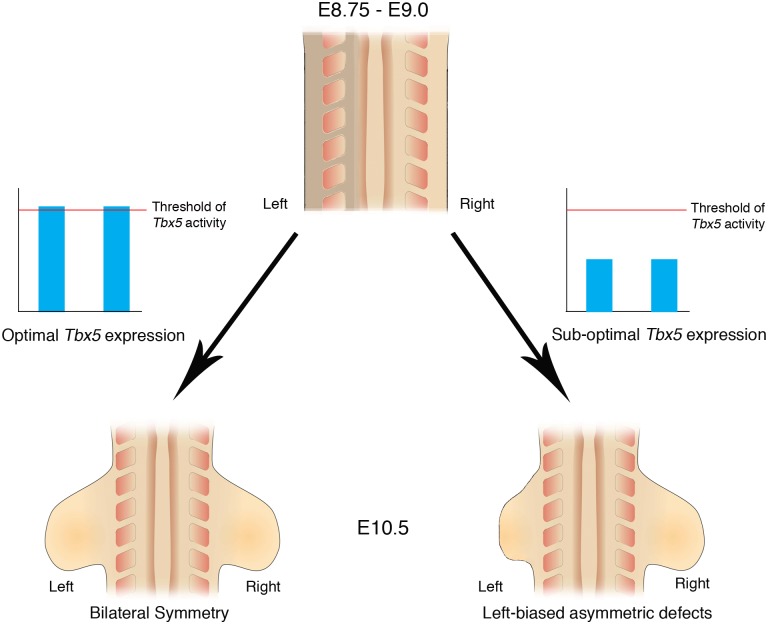
Schematic representation of *Tbx5* buffering the inherent LPM L/R asymmetry. Bilateral optimal *Tbx5* expression reaches above a threshold level that buffers the inherent asymmetry in the left and right LPM, which includes the future forelimb-forming regions. Bilateral, suboptimal *Tbx5* expression levels that fail to reach threshold levels leads to abnormalities in limb formation and cannot buffer the inherent asymmetry in the LPM and as a result the left limb is more severely affected by lowered Tbx5 activity than the right limb.

Here, we demonstrate that inherent asymmetry between the left and right LPM is buffered by threshold levels of Tbx5 that ensure bilaterally symmetric forelimb formation (Fig 7).

### Bilaterally symmetric limb development is not the ground state

The simplest explanation for how bilateral symmetry in limb formation is achieved is by default and that the gene programmes controlling limb formation can operate with equal fidelity on the left and right sides of the embryo. Over the last 20 years however, it has become clear that the pathway that breaks L/R symmetry in the very early embryo and ultimately establishes the asymmetry in visceral organs has an impact on the LPM. Components of the L/R pathway, *Nodal*, *Lefty* and *Pitx2* are expressed in the left LPM at stages prior to limb formation, however, the effects of these genes on bilateral symmetry of the limb have not been appreciated, previously. Our results indicate that bilaterally symmetric limb development is not the ground state but instead the ‘memory’ of L/R pathway genes expressed in the left LPM can interfere with the ability of LPM cells to establish *Fgf10*-*Fgf8* positive feedback loop essential for limb outgrowth, and that threshold levels of *Tbx5* are required to buffer this developmental history. Furthermore, we demonstrate that one direct target of *Tbx5*, *Fgf10*, does not have the ability to mask the bilateral asymmetry, suggesting that *Tbx5* carries out this buffering role upstream of *Fgf10*.

Other bilaterally symmetric structures in the embryo, such as the somites, also need to override the influence of early asymmetric gene expression associated with the L/R pathway. RA signalling is essential to synchronize somite formation between left and right sides by antagonizing Fgf8 to ensure symmetric FGF signalling activity on the both sides of embryos [26–28]. Together with our study, these results suggest that buffering the effects of the left-right pathways is a general strategy employed during the formation of bilaterally symmetric structures in the developing embryo. Significantly, however, the mechanisms by which bilateral symmetry is achieved are different in different structures, such as the limbs and somites. During somitogenesis, the L/R asymmetric signals are counter-balanced by asymmetric expression of *Nr2f2*, a nuclear receptor that promotes the transcriptional activity of the RA signaling pathway [29]. In contrast, in the limb, bilaterally symmetric *Tbx5* expression at levels above a threshold are able to buffer the effects of the L/R pathway. A buffering mechanism that operates through bilaterally symmetric gene expression may be more robust than one that relies on establishing asymmetric gene expression to counterbalance a ‘historic’ gene expression asymmetry.

### Molecular mechanisms buffering bilateral limb asymmetry

While *Tbx* hypomorph mutants and HOS patients are unique in that they display left-biased forelimb defects, a few other mutant mice with oriented asymmetric forelimb outgrowth defects have been reported [21,30–32]. *Retinaldehyde dehydrogenase 2* (*Raldh2*) mutants rescued by maternal administration of RA display more severely affected left forelimbs than right [32], similar to *Tbx* hypomorph mutants. Furthermore, the defects in these rescued forelimbs include triphalangeal thumbs, lack of thumbs and loss of humerus as seen in *Tbx* hypomorph mutants. Since RA signalling directly regulates *Tbx5* transcription [33], *Tbx5* expression may be at hypomorphic levels in these rescued embryos, which would explain the left-biased forelimb defects.

Two transgene insertion mutant mice, *legless* and *footless*, display right-biased forelimb defects [21,30,31]. The *Legless* mutation causes hypomorphic expression of *Sp8*, a zinc finger transcription factor expressed in the AER [34], and the *footless* mutation causes hypomorphic expression of *R-spondin 2*, a secreted protein also expressed in the AER [35]. Since these genes are both co-expressed in AER and *Sp8* is required for *R-spondin2* expression, hypomorphic levels of *Sp8* in the *legless* mutant is likely the cause of hyopmorphic levels of *R-spondin2*. Although how hypomorphic levels of *R-spondin2* causes the forelimb asymmetry is not understood, this mutant demonstrates that bilateral disruption of the Wnt pathway can produce right-biased asymmetric forelimb defects. Further studies will reveal how input from different signaling pathways is buffered to ensure that their ultimate output, in the form of limb structures is bilaterally symmetric.

Asymmetrical limb defects can be induced by teratogens such as cadmium, acetazolamide, MNNG and acetoxymethyl-methylnitrosamine [36–41]. The mechanisms causing these asymmetrical defects are not understood however. The affected mice do not show anterior biased limb defects or other clinical features of HOS as seen in *Tbx5* hypomorph mutants suggesting these teratogens are not acting by disrupting the *Tbx5* pathway or act on only one component of what could be several different pathways regulated by *Tbx5*.

### Left-bias severity of HOS limb defects

The consensus is that HOS is caused by haploinsufficiency rather than pathogenic mutations producing dominant-negative acting forms of the protein. HOS pathogenic mutations have been identified throughout the coding sequence and are not exclusively localized to either the DNA binding T domain or regions thought to interact with cofactors. Functional studies of mutations that lie within the T domain show these forms of the protein have reduced DNA binding ability and diminished binding with interaction partners consistent with these mutations leading to reduced transcriptional activation of target genes leading to functional haploinsufficiency [42].

HOS is unique among congenital abnormalities affecting the limb in that a consistent feature of the clinical presentation is the left-biased severity in the defects [3]. Our results show that the asymmetry in the severity of limb phenotype results as a consequence of an effect on the very earliest events of limb bud formation when the precursors of limb structures are recruited. These events are sensitive to the inherent asymmetry in the limb-forming LPM when sub-threshold, hypomorphic levels of Tbx proteins are present. Our analysis of the penetrance in genetically defined HOS patients and our observations in our HOS mouse model indicate left-biased defects present with almost 100% penetrance. Our results provide an explanation for origins of the left-biased severity of HOS defects and confirm it as key a diagnostic criterion to indicate HOS.

Shoulder/girdle involvement is a cardinal feature of HOS and the humerus can also be affected and this is reflected in our mouse mutants. Overall, we observed more severe shoulder and humerus defects in our mouse mutant series than have been described in HOS patients and this may reflect the generally more severe severity range in limb defects we observe in our mouse mutant compared to HOS. In clinical descriptions of HOS, greater emphasis has been placed on description of more distal structures such as radius and hand that are expected to have greater impact on patient limb function. Shoulder girdle and humerus abnormalities are often poorly described making it difficult to compare to the defects we see in the mouse.

## Materials and Methods

### Ethics statement

The electronic patient record at Great Ormond Street was reviewed and approved by the hospital clinical audit committee (ref 1337). Data were extracted into a linked anonymised database. Animal work was carried out under an appropriate Home Office licence and approved by the local ethics panel (AWERB).

### Embryos and mouse lines

Mice were staged according to Kaufman 2001 [43]. Noon on the day a vaginal plug was observed was taken to be 0.5 embryonic days (E) of development. *Prx1Cre* [9], *Prx1Cre(98)* [16], conditional *Tbx5* [44], *INV* [7], *Rosa26RLacZ* [45] and the chimeric transgenic lines [8] have all been described previously. The *Z/EGFgf10* line was produced using the *Z/EG* backbone [46] provided by C. Lobe. Briefly a mouse *Fgf10* cDNA was inserted into the Z/EG construct upstream of the IRESeGFP cassette. The full construct was transfected into ES cells. Cells that had successfully integrated the construct were selected with G418, screened for single integration events by Southern Blott and surviving clones were used to generate chimeras from which founder animals were derived.

### Whole mount *in situ* hybridisation

Whole mount *in situ* hybridisation protocols were carried out as previously described [47]. A minimum of 4 mutant embryos were analysed for each genotype at each stage. *mFgf10* [48] and *mFgf8* [49] probes were reported previously. We used a full-length mouse *Sall4* clone as a probe template.

### Skeletal preparations

E14.5 and E17.5 embryo skeletons were stained using alizarin red (for bone) and alcian blue (for cartilage) [50].

### Quantitative PCR

RNA was extracted from the 10 forelimb buds of embryos according to the manufacturer’s instructions using the RNeasy mini kit (Qiagen) and cDNA was subsequently prepared by using SuperScript III Reverse Transcriptase (Invitrogen).

Primers were generated using the PrimerBlast application available online (http://www.ncbi.nlm.nih.gov/tools/primer-blast). The following primer pairs were used: *Cre recombinase* Fwd 5’- GAACGAAAACGCTGGTTAGC -3’ Rev 5’- CCCGGC AAAACAGGTAGTTA -3’, *Prx1-Tbx* Fwd 5’- GAGACAGCTTTTATCGCTGTG -3’ Rev 5’- CATCGCTGCCCCGGAATCCCT -3’, *GAPDH* Fwd 5’- TGTCAGCAATGCATCCTGCA -3’ Rev 5’- CCGTTCAGCTCTGGGATG AC -3’. The two-tailed Student’s *t*-test was used for statistical analysis.

### Western blot analysis

Wild type and mutant forelimb buds (E10.5) were homogenized in RIPA buffer. A rabbit polyclonal Tbx5 antibody raised against a peptide spanning an N-terminal region of the protein was used (details are available on request). Densitometry was performed by scanning the original films and then analyzing the bands with ImageJ (NIH).

### Analysis of HOS patients

The electronic patient record at Great Ormond Street was searched for the terms ‘Tbx5’ and ‘Holt-Oram’. Data on the cardiac, genetic and upper limb changes of affected patients were extracted by G.M. into a linked anonymised database. Patients without recorded *Tbx5* mutation analysis were cross-checked with the national reference laboratory in Nottingham, and missing results retrieved. This review was approved by the hospital clinical audit committee (ref 1337).

## Supporting Information

S1 Fig Generation of Tbx hypomorphic mutant mouse. **A,** A schematic representation of a chimeric construct of N-terminus and T-domain of *Tbx5* (green) fused to C-terminus of *Tbx4* (blue). **B-E,** Western blot for Tbx5 and actin loading control from E10.5 control and *Tbx5*^*lox/lox*^*;Prx1Cre;Prx1-Tbx* mutant limbs (B) and E10.5 *Tbx5* ^*+/+*^*;Prx1Cre* and *Tbx5* ^*lox/+*^*;Prx1Cre* mutant limbs (D). The relative density of Tbx5 protein bands was normalized with actin (C and E).
(TIF)

S2 Fig Cre is active at equivalent levels in the left and right forelimbs in the *Prx1Cre(98)* line. LacZ staining of *Rosa26RLacZ/+;Prx1Cre(98)* embryos at E10.5 (A and B), E10.75 (C and D) and E11.5 (E and F).
(TIF)

S3 Fig *Tbx5*^*lox/lox*^*;Prx1Cre;Prx1-Tbx;INV/INV* embryos with *situs inversus* show right biased forelimb defects. **A-B,** *Tbx5*^*lox/lox*^*;Prx1Cre;Prx1-Tbx;INV/INV* embryo with *situs solitus* at E15.5. The left forelimb (B) is more severely affected than the right forelimb (A). The right forelimb has 4 digits (A), while the left forelimb has three digits (B). **C-F,** *Tbx5*^*lox/lox*^*; Prx1Cre;Prx1-Tbx;INV/INV* embryos with *situs inversus* at E15.5 (C and D) or E12.5 (E and F). The right forelimb (C and E) is more severely affected than the left forelimb (D and F) in both embryos. The right forelimb is absent (C) while the left forelimb has two digits (D). The right forelimb bud (E) is smaller than the left one (F).
(TIF)

S4 Fig Delivery of transgenic *Fgf10* expression in the forelimb LPM is sufficient to rescue forelimb outgrowth defects in *Fgf10* null mutant embryos. **A-C,** Skeletal preparation of E17.5 control embryo (B) and forelimbs (C). **D-F,** *Fgf10*^*-/-*^ null mutant embryo forms only rudimentary scapula elements on both right and left sides (arrow). **G-I,** *Fgf10*^*-/-*^*;Prx1Cre;Z/EGFgf10* mutant embryo forms normal forelimbs (arrow), suggesting that expression of *Fgf10* from *Z/EGFgf10* is sufficient to rescue the limb outgrowth defects caused by the lack of *Fgf10* gene.
(TIF)

S1 Table Grading of HOS patients limb defects. The defects in the left and right limbs of 8 HOS patients were graded using the Blauth and Bayne and Klug systems [10–13] and tabulated. Pathogenic mutations of *TBX5* are confirmed for all patients. 4 Patients (1,4,5,11) have more severe scores in the left limb compared to right. 4 Patients (2,3,6,10) have bilateral Blauth and Bayne & Klug scores. * For patients 3,6,10, the overall severity of the limb defects were scored on additional features of their clinical presentation. *^1^, from direct measurements of X-rays, the left thumb is shorter than the right (L = 40.9mm, R = 45.1mm, measured from distal phalangeal tip to MCPj), *^2^ phenotypic description in patients notes produced by assessing consultant geneticist at GOSH which explicitly describes a left-sided bias in limb defects, *^3^ the right limb has proximal radioulnar synostosis while the left limb has proximal and distal radioulnar synostosis.
(DOCX)

S2 Table Numbers of asymmetrical and symmetrical limb defects observed in the mutant embryos analysed. Fisher’s exact test was used (* p<0.05, ** p<0.01, n.s.; not significant). *Tbx5*^*lox/lox*^*;Prx1Cre;Prx1-Tbx* mutant (row 2), *Tbx5*^*lox/lox*^*;Prx1Cre(98)* (row 4) and *Fgf10-/-;Prx1Cre;Z/EGFgf10* (row 6) were compared with wild type (row 1) (shown in black). *Tbx5*^*lox/lox*^*;Prx1Cre;Prx1-Tbx;INV/INV* mutant (row 3) and *Tbx5*^*lox/lox*^*;Prx1Cre;Prx1-Tbx;Z/EGFgf10* (row 5) were compared with *Tbx5*^*lox/lox*^*;Prx1Cre;Prx1-Tbx* mutant (row 2) (shown in red).
(DOCX)
