# Supplementary figures and images for: Tbx5 Buffers Inherent Left/Right Asymmetry Ensuring Symmetric Forelimb Formation
Source: PLoS Genet. 2016 Dec 19;12(12):e1006521. doi: 10.1371/journal.pgen.1006521 (PMC5215935; doi:10.1371/journal.pgen.1006521)

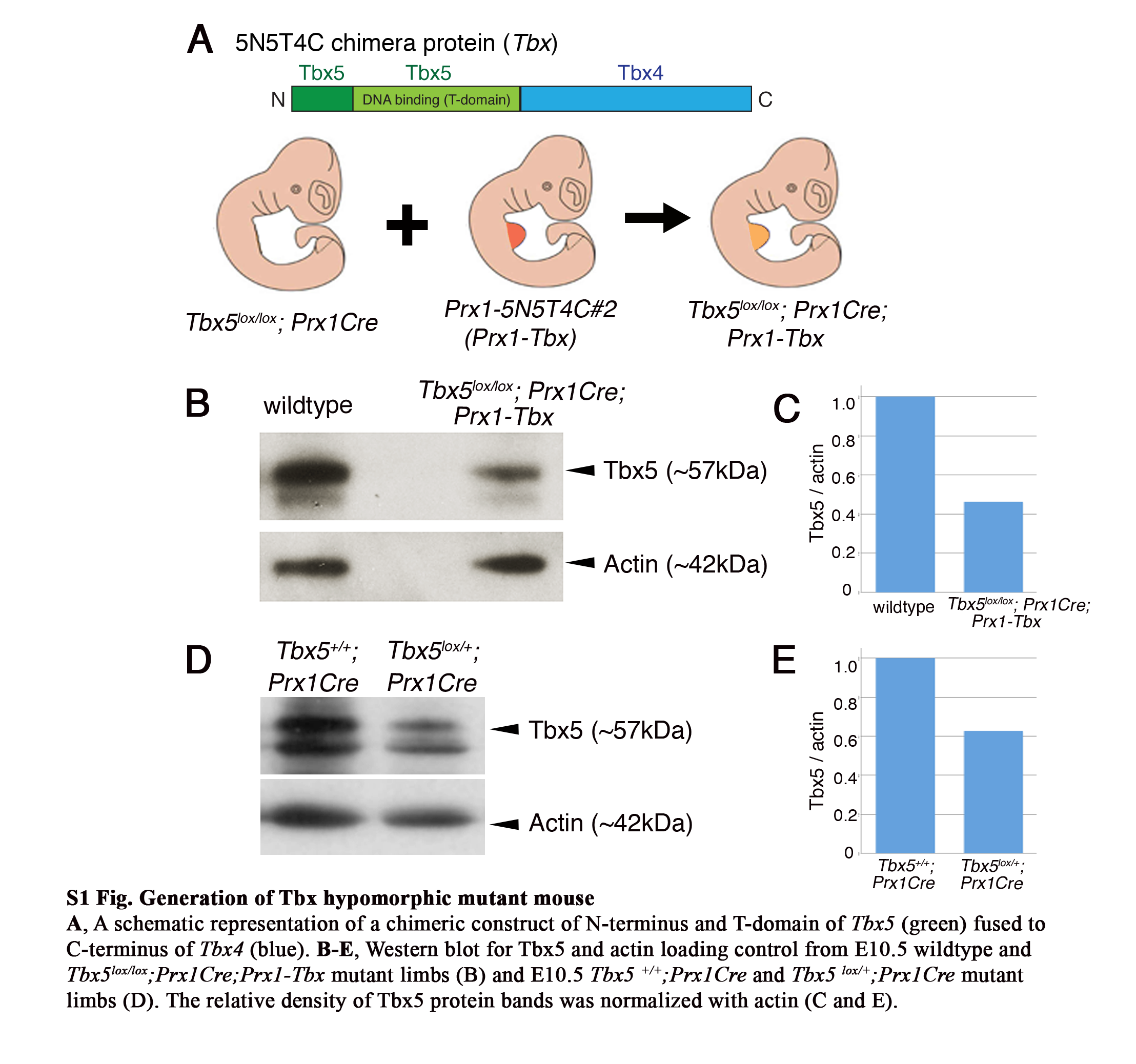

Supplement: S1 Fig — A, A schematic representation of a chimeric construct of N-terminus and T-domain of Tbx5 (green) fused to C-terminus of Tbx4 (blue). B-E, Western blot for Tbx5 and actin loading control from E10.5 control and Tbx5lox/lox;Prx1Cre;Prx1-Tbx mutant limbs (B) and E10.5 Tbx5 +/+;Prx1Cre and Tbx5 lox/+;Prx1Cre mutant limbs (D). The relative density of Tbx5 protein bands was normalized with actin (C and E). (TIF) [file pgen.1006521.s001.tif]

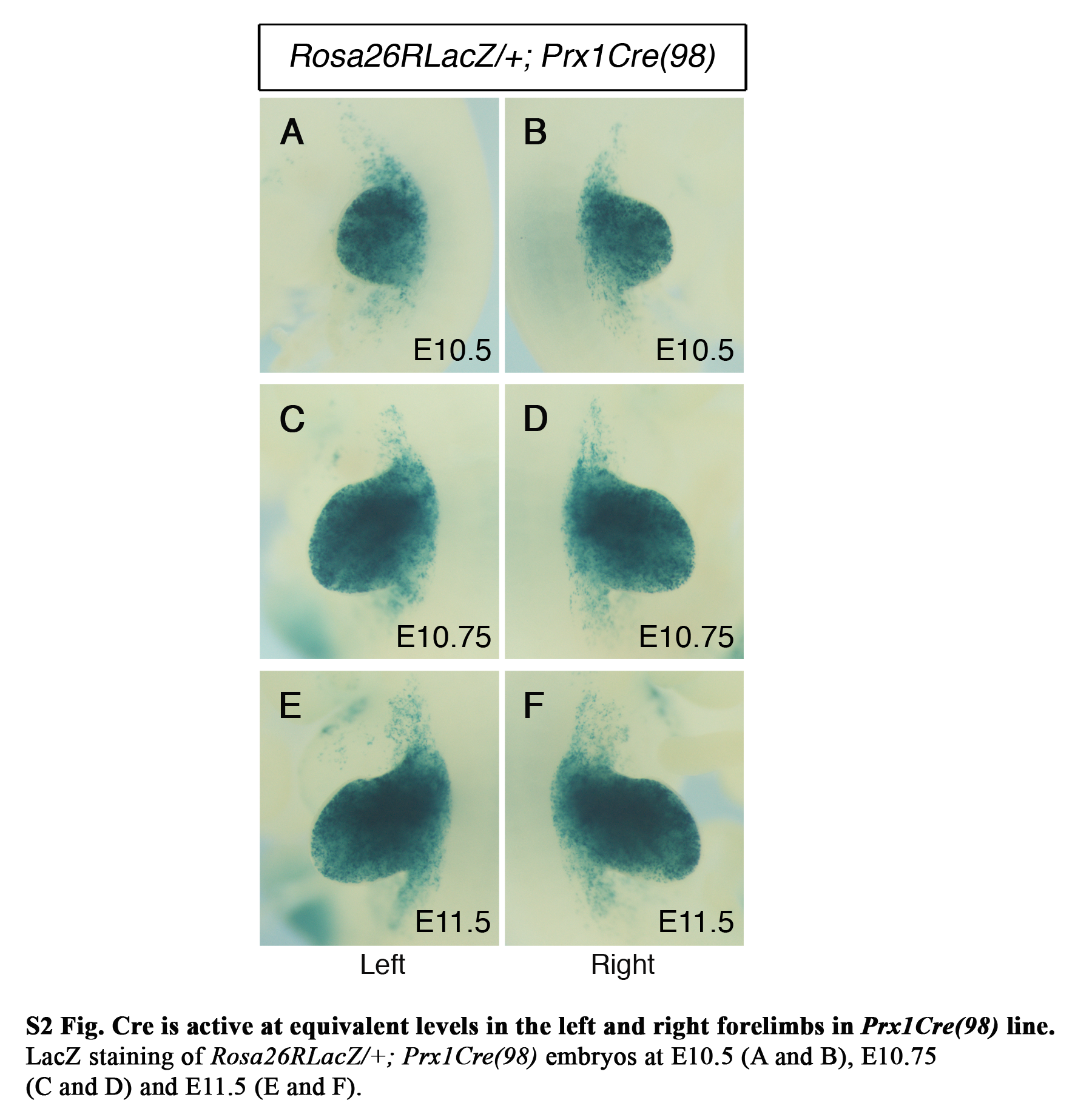

Supplement: S2 Fig — LacZ staining of Rosa26RLacZ/+;Prx1Cre(98) embryos at E10.5 (A and B), E10.75 (C and D) and E11.5 (E and F). (TIF) [file pgen.1006521.s002.tif]

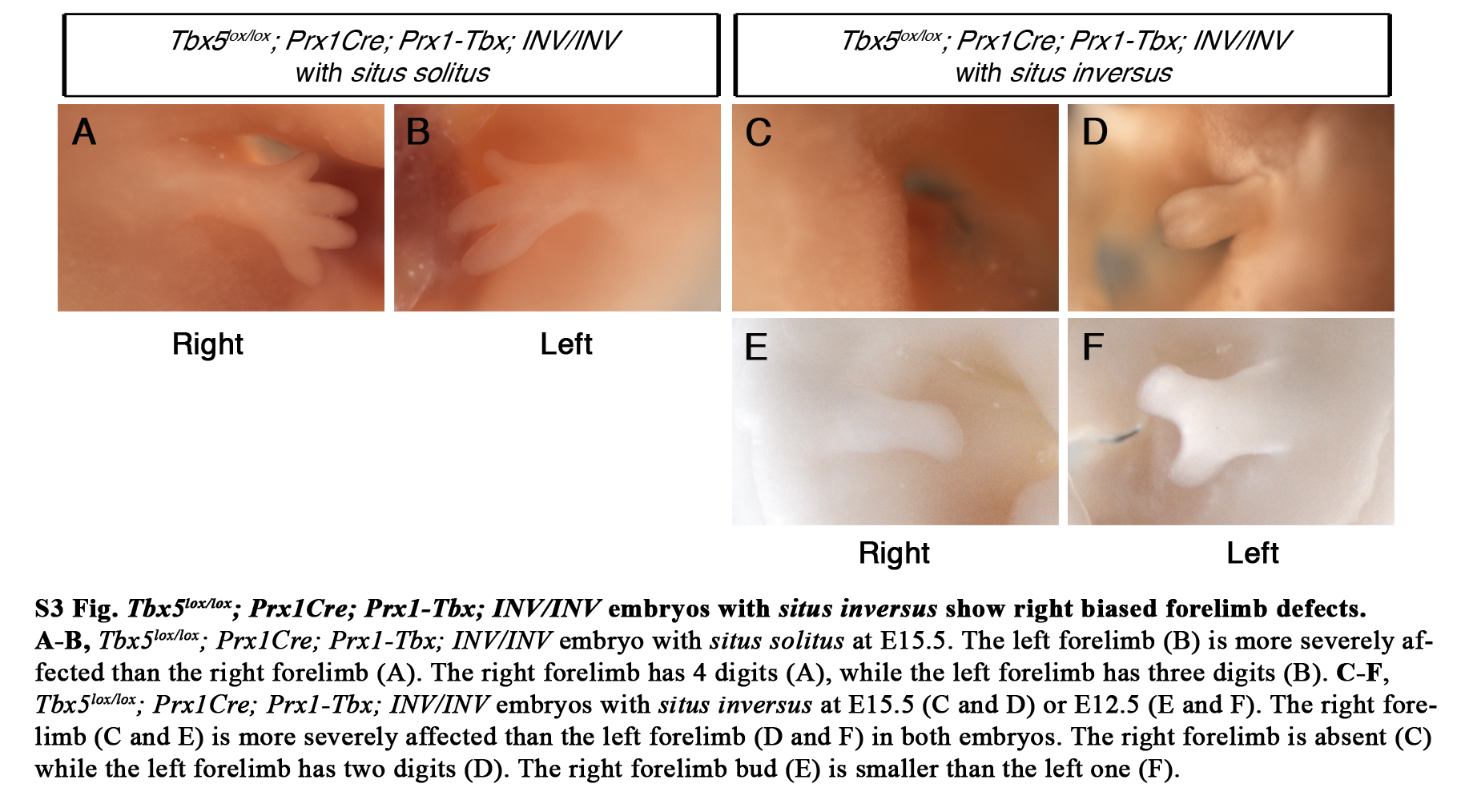

Supplement: S3 Fig — A-B, Tbx5lox/lox;Prx1Cre;Prx1-Tbx;INV/INV embryo with situs solitus at E15.5. The left forelimb (B) is more severely affected than the right forelimb (A). The right forelimb has 4 digits (A), while the left forelimb has three digits (B). C-F, Tbx5lox/lox; Prx1Cre;Prx1-Tbx;INV/INV embryos with situs inversus at E15.5 (C and D) or E12.5 (E and F). The right forelimb (C and E) is more severely affected than the left forelimb (D and F) in both embryos. The right forelimb is absent (C) while the left forelimb has two digits (D). The right forelimb bud (E) is smaller than the left one (F). (TIF) [file pgen.1006521.s003.tif]

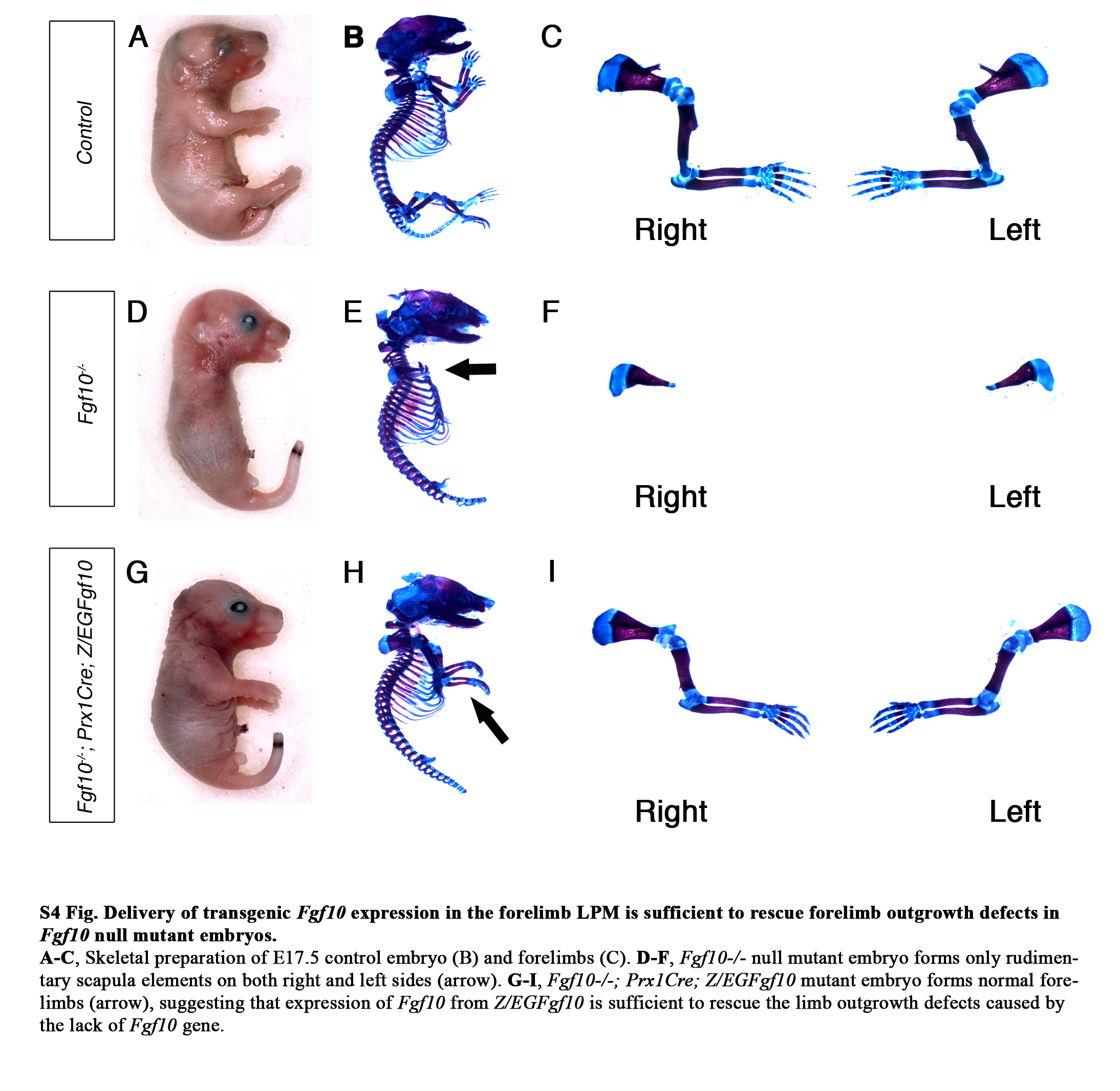

Supplement: S4 Fig — A-C, Skeletal preparation of E17.5 control embryo (B) and forelimbs (C). D-F, Fgf10-/- null mutant embryo forms only rudimentary scapula elements on both right and left sides (arrow). G-I, Fgf10-/-;Prx1Cre;Z/EGFgf10 mutant embryo forms normal forelimbs (arrow), suggesting that expression of Fgf10 from Z/EGFgf10 is sufficient to rescue the limb outgrowth defects caused by the lack of Fgf10 gene. (TIF) [file pgen.1006521.s004.tif]
