# Supplementary material for: Tbx5 Buffers Inherent Left/Right Asymmetry Ensuring Symmetric Forelimb Formation
Source: PLoS Genet. 2016 Dec 19;12(12):e1006521. doi: 10.1371/journal.pgen.1006521 (PMC5215935; doi:10.1371/journal.pgen.1006521)
Supplement: S1 Table — The defects in the left and right limbs of 8 HOS patients were graded using the Blauth and Bayne and Klug systems [10–13] and tabulated. Pathogenic mutations of TBX5 are confirmed for all patients. 4 Patients (1,4,5,11) have more severe scores in the left limb compared to right. 4 Patients (2,3,6,10) have bilateral Blauth and Bayne & Klug scores. * For patients 3,6,10, the overall severity of the limb defects were scored on additional features of their clinical presentation. *1, from direct measurements of X-rays, the left thumb is shorter than the right (L = 40.9mm, R = 45.1mm, measured from distal phalangeal tip to MCPj), *2 phenotypic description in patients notes produced by assessing consultant geneticist at GOSH which explicitly describes a left-sided bias in limb defects, *3 the right limb has proximal radioulnar synostosis while the left limb has proximal and distal radioulnar synostosis. (DOCX) [file pgen.1006521.s005.docx]

| Patient No | Left | | Right | |  | Genetic Mutation of TBX5 | Cardiac position |
| --- | --- | --- | --- | --- | --- | --- | --- |
|  | Thumb | Radius | Thumb | Radius |  |  |  |
| 1 | Blauth V | Type II | Blauth IV | Type II | Left biased | 239 G>A (Gly80Glu in T-box domain) | *situs solitus* |
| 2 | Blauth V | Type II | Blauth V | Type II | Symmetrical | Deletion of Ile172-Ile222 | *situs solitus* |
| 3 | Blauth IIa | Type I | Blauth IIa | Type I | Left biased*^1^ | Deletion of exon3-8 | *situs solitus* |
| 4 | Blauth V | Type IV | Blauth II | N | Left biased | 874 C>T (premature termination) | *situs solitus* |
| 5 | Blauth V | Type IV | Blauth IV | Type III | Left biased | Deletion of exon1-10 | *situs solitus* |
| 6 | Blauth V | Type IV | Blauth V | Type IV | Left biased*^2^ | 443 G>A (premature termination) | *situs solitus* |
| 10 | Blauth V | Type I | Blauth V | Type I | Left biased*^3^ | Insertion of A at 593-594 (premature termination) | *situs solitus* |
| 11 | Blauth IIb | Type I | Blauth IIa | N | Left biased | Deletion of 1044 T (premature termination) | *situs solitus* |

|  |  |  |  |
| --- | --- | --- | --- |
|  | **Thumb Hypoplasia (modified Blauth system)** | |  |
|  | I | Mild hypoplasia, all structures present |  |
|  | IIa | Moderate hypoplasia (thenar muscles, 1st web space tight). Lax UCL at MCPj |  |
|  | IIb | As IIa, but globally unstable MCPj |  |
|  | IIIa | Severe hypoplasia, including metacarpal. Relatively stable CMCj |  |
|  | IIIb | As IIIa, but unstable CMCj |  |
|  | IV | No metacarpal - 'floating thumb' |  |
|  | V | Complete absence |  |
|  |  |  |  |
|  |  |  |  |
|  | **Radial dysplasia (Bayne & Klug)** | |  |
|  | I | Short distal radius |  |
|  | II | Hypoplastic radius, but proximal & distal epiphyses present |  |
|  | III | Partial absence of radius (typically distal 1-2 thirds) |  |
|  | IV | Complete absence of radius |  |
|  |  |  |  |

**S1 Table. Grading of HOS patients limb defects**

The defects in the left and right limbs of 8 HOS patients were graded using the Blauth and Bayne and Klug systems [[10-13](#_ENREF_10)] and tabulated. Pathogenic mutations of *TBX5* are confirmed for all patients. 4 Patients (1,4,5,11) have more severe scores in the left limb compared to right. 4 Patients (2,3,6,10) have bilateral Blauth and Bayne & Klug scores. * For patients 3,6,10 the overall severity of the limb defects were scored on additional features of their clinical presentation. *1, from direct measurements of X-rays, the left thumb is shorter than the right (L=40.9mm, R=45.1mm, measured from distal phalangeal tip to MCPj), *^2^ phenotypic description in patients notes produced by assessing consultant geneticist at GOSH which explicitly describes a left-sided bias in limb defects, *^3^ the right limb has proximal radioulnar synostosis while the left limb has proximal and distal radioulnar synostosis.
