# Supplementary material for: Tbx5 Buffers Inherent Left/Right Asymmetry Ensuring Symmetric Forelimb Formation
Source: PLoS Genet. 2016 Dec 19;12(12):e1006521. doi: 10.1371/journal.pgen.1006521 (PMC5215935; doi:10.1371/journal.pgen.1006521)
Supplement: S2 Table — Fisher’s exact test was used (* p<0.05, ** p<0.01, n.s.; not significant). Tbx5lox/lox;Prx1Cre;Prx1-Tbx mutant (row 2), Tbx5lox/lox;Prx1Cre(98) (row 4) and Fgf10-/-;Prx1Cre;Z/EGFgf10 (row 6) were compared with wild type (row 1) (shown in black). Tbx5lox/lox;Prx1Cre;Prx1-Tbx;INV/INV mutant (row 3) and Tbx5lox/lox;Prx1Cre;Prx1-Tbx;Z/EGFgf10 (row 5) were compared with Tbx5lox/lox;Prx1Cre;Prx1-Tbx mutant (row 2) (shown in red). (DOCX) [file pgen.1006521.s006.docx]

| genotype | Left Biased | Right Biased | Symmetrical |
| --- | --- | --- | --- |
| wild type | 0 | 0 | 15 |
| *Tbx5^lox/lox^; Prx1Cre; Prx1-Tbx* ** | 11 | 0 | 0 |
| *Tbx5^lox/lox^;Prx1Cre;Prx1-Tbx;INV/INV showing situs inversus* * | 0 | 3 | 0 |
| *Tbx5^lox/lox^;Prx1Cre (98)* ** | 18 | 0 | 0 |
| *Tbx5^lox/lox^;Prx1Cre;Prx1-Tbx;Z/EGFgf10* ^n.s.^ | 4 | 0 | 0 |
| *Fgf10^-/-^;Prx1Cre;Z/EGFgf10* ^n.s.^ | 0 | 0 | 2 |

**S2 Table. Numbers of asymmetrical and symmetrical limb defects observed in the mutant embryos analysed.**

Fisher’s exact test was used (* p<0.05, ** p<0.01, n.s.; not significant). *Tbx5^lox/lox^;Prx1Cre;Prx1-Tbx* mutant (row 2), *Tbx5^lox/lox^;Prx1Cre(98)* (row 4) and *Fgf10-/-;Prx1Cre;Z/EGFgf10* (row 6) were compared with wild type (row 1) (shown in black). *Tbx5^lox/lox^;Prx1Cre;Prx1-Tbx;INV/INV* mutant (row 3) and *Tbx5^lox/lox^;Prx1Cre;Prx1-Tbx;Z/EGFgf10* (row 5) were compared with *Tbx5^lox/lox^;Prx1Cre;Prx1-Tbx* mutant (row 2) (shown in red).
